# Predicting Ischemic Stroke in Acute Coronary Syndrome Patients: A Machine Learning Approach Using Electronic Medical Records

**DOI:** 10.7759/cureus.72258

**Published:** 2024-10-24

**Authors:** Faishal Hanif, Mawaddah A Rochmah, Ismail Setyopranoto, Abdul Gofir, Paryono Paryono, Lukman A Chandra, Ajeng V Icanervilia, Yudha N Patria, Vitayanti Anggraeni, Dhite B Nugroho

**Affiliations:** 1 Department of Neurology, Faculty of Medicine, Universitas Jenderal Soedirman/Prof. Dr. Margono Soekarjo Hospital, Purwokerto, IDN; 2 Department of Neurology, Faculty of Medicine, Public Health, and Nursing, Universitas Gadjah Mada, Yogyakarta, IDN; 3 Department of Pharmacology and Therapy, Faculty of Medicine, Public Health, and Nursing, Universitas Gadjah Mada, Yogyakarta, IDN; 4 Department of Radiology, Faculty of Medicine, Public Health, and Nursing, Universitas Gadjah Mada, Yogyakarta, IDN; 5 Department of Pediatrics, Faculty of Medicine, Public Health, and Nursing, Universitas Gadjah Mada, Yogyakarta, IDN; 6 Department of Internal Medicine, Faculty of Medicine, Public Health, and Nursing, Universitas Gadjah Mada, Yogyakarta, IDN

**Keywords:** acute coronary syndrome, ischemic stroke, machine learning, predictive modeling, prognosis

## Abstract

Background

Acute coronary syndrome (ACS) is a leading cause of morbidity and mortality worldwide. Despite advances in management, patients with ACS remain at a significant risk of developing ischemic stroke (IS), a serious complication associated with high mortality and long-term disability. The accurate prediction of stroke risk in ACS patients can facilitate timely interventions and improve clinical outcomes.

Objective

This study aimed to develop and validate machine learning (ML) models to predict ischemic stroke within one year of ACS diagnosis, using electronic medical records (EMRs) from a tertiary care hospital in Indonesia.

Methods

We conducted a retrospective cohort study using data from 4,789 ACS patients treated at Dr. Sardjito Hospital between 2018 and 2022. Machine learning models, including Logistic Regression, Random Forest, and XGBoost, were trained and validated using patient demographics, comorbidities, and clinical variables. Model performance was assessed using precision, accuracy, sensitivity, specificity, and area under the curve (AUC)-receiver operating characteristic (ROC).

Results

Among the study cohort, 212 patients (4.4%) developed ischemic stroke within one year. Logistic Regression demonstrated a balanced performance with a sensitivity of 65%, a specificity of 70%, and an AUC-ROC of 0.70. Random Forest and XGBoost models achieved higher sensitivities (94% and 95%, respectively) but had lower specificities (12% each). The most significant predictors of ischemic stroke included ST-segment elevation myocardial infarction (STEMI), age of ≥60 years, atrial fibrillation, hypertension, and chronic kidney disease.

Conclusion

The Logistic Regression model, with its balanced sensitivity and specificity, offers a reliable tool for predicting ischemic stroke in ACS patients. The implementation of this model in clinical practice could enhance risk stratification and inform personalized treatment strategies. Future studies should focus on prospective validation and the integration of additional clinical variables.

## Introduction

Noncommunicable diseases (NCDs), particularly cardiovascular diseases (CVDs) and strokes, have become a significant global health concern, accounting for approximately 74% of deaths worldwide [1]. The World Health Organization (WHO) reports that ischemic heart disease and stroke are responsible for nearly 18 million deaths annually, with a substantial burden falling on low- and middle-income countries [2]. The increasing prevalence of risk factors such as hypertension, diabetes, and obesity, alongside an aging population, is expected to exacerbate this situation [3].

Acute coronary syndrome (ACS), a critical manifestation of ischemic heart disease, includes unstable angina and myocardial infarction (MI), resulting from sudden reductions in blood flow to the heart. Despite advancements in treatment, patients with ACS remain at high risk for complications, including recurrent MI, heart failure, and stroke [4]. Specifically, the cumulative incidences of ischemic stroke (IS) were 1.0% in the first month, 1.9% in the first year, and 9.0% at 13 years after ACS [5]. The pathophysiology of stroke in this context is complex and involves factors such as atrial fibrillation and left ventricular dysfunction, which complicate patient management and increase the need for effective risk stratification [5].

Traditional models for stroke prediction, such as the Global Registry of Acute Coronary Events (GRACE) and CHA2DS2-VASc scores, may result in suboptimal outcomes, particularly in the ACS population, where stroke risk is influenced by a multitude of interrelated factors [6]. This inadequacy highlights the necessity for more sophisticated predictive tools. Recent studies indicate that machine learning (ML) approaches can enhance predictive modeling by analyzing large datasets and identifying complex, nonlinear relationships among variables [7]. ML models can adapt to new data inputs, refining their predictive capabilities, which is particularly beneficial in dynamic clinical environments [8].

The application of ML in healthcare has shown promise across various domains, including image analysis and predictive analytics [9]. In the context of ACS, ML-based models have the potential to integrate diverse data sources, such as demographic information, clinical parameters, laboratory results, and imaging data, to more accurately identify patients at risk of ischemic stroke [10]. This capability allows for improved risk stratification and the development of personalized treatment plans, which may include more aggressive anticoagulation therapy and closer monitoring.

The increasing burden of NCDs, particularly CVDs and strokes, necessitates the development of more accurate predictive models for managing patients with ACS. By employing machine learning techniques, this study seeks to advance the current state of risk prediction for ischemic stroke, laying the groundwork for future research and clinical applications in cardiovascular care.

The primary objective of this study is to develop and validate a machine learning-based predictive model for ischemic stroke in patients with acute coronary syndrome, utilizing electronic medical records (EMRs) from a large tertiary care hospital. By leveraging the comprehensive data available in EMRs, this study aims to create a predictive tool that can be integrated into clinical workflows, thereby enhancing decision-making and improving patient outcomes.

## Materials and methods

Study design

This study employed a retrospective cohort design, utilizing EMRs from Dr. Sardjito Hospital, a tertiary care hospital in Yogyakarta, Indonesia. The study period spanned from January 2018 to December 2022, allowing for the inclusion of comprehensive data on ACS patients over a five-year period. The objective was to develop and validate a predictive model for ischemic stroke occurring within one year following an ACS event, using advanced machine learning techniques. All analyses were performed using R (R Foundation for Statistical Computing, Vienna, Austria), a widely used environment for statistical computing, with packages such as tidymodels, vip, and shapper for machine learning modeling and interpretation [11]. This study was approved by the Medical and Health Research Ethics Committee (MHREC) of Universitas Gadjah Mada, with approval number KE/FK/1405/EC/2023. Ethical considerations were strictly adhered throughout the research process.

Population

The study population included patients diagnosed with ACS, which encompasses ST-segment elevation myocardial infarction (STEMI), non-ST-segment elevation myocardial infarction (NSTEMI), and unstable angina. These diagnoses were identified based on the International Classification of Diseases, Tenth Revision (ICD-10) codes, including I20.0, I20.9, I21.0, I21.1, I21.2, I21.3, I21.4, I21.9, I22.0, I22.1, I22.2, I22.8, and I22.9. The inclusion criteria required patients to be aged 18 years or older at the time of diagnosis and to have complete follow-up data for at least one year post-ACS. Patients were excluded if they had a history of ischemic stroke prior to the ACS event, as indicated by ICD-10 codes I63.0 and I64.0. Additionally, patients who did not have any follow-up visits or were lost to follow-up immediately after the initial ACS diagnosis were excluded from the study. Finally, any patients with missing or incomplete data for key variables required for model development were not included in the analysis.

Data collection

Data were meticulously extracted from the EMRs of Dr. Sardjito Hospital, focusing on a comprehensive set of variables that are relevant to both ischemic stroke risk and ACS outcomes. The data collection encompassed several key categories. Demographic variables included patient age and gender, and clinical characteristics recorded for each patient comprised the type of ACS, whether STEMI, NSTEMI, or unstable angina. In addition to these clinical and demographic factors, the study also collected data on comorbidities, which were identified using specific ICD-10 codes. These included atrial fibrillation (ICD-10: I48), hypertension (ICD-10: I10, I11.0, and I11.9), diabetes mellitus (ICD-10: E11.0-E11.9), dyslipidemia (ICD-10: E78.0-E78.8), and chronic kidney disease (ICD-10: N17.9). Procedural data were also collected, detailing coronary interventions such as percutaneous coronary intervention (PCI) and coronary artery bypass grafting (CABG). The length of hospital stay and any rehospitalizations within one year were also documented to provide a comprehensive overview of each patient's post-ACS outcomes.

Machine learning methods

Data Splitting and Recipe Preparation

After preprocessing, the data were split into training and testing sets to prepare for model training and evaluation. A 75/25 split was implemented using the initial_split() function, with 75% of the data allocated to the training set and 25% to the testing set. Stratification was performed based on the outcome variable (case) to ensure balanced classes in both the training and testing sets.

To preprocess the training data, a recipe was prepared using the tidymodels package. The recipe included several key steps: handling novel levels (novel levels in categorical data were managed using step_novel()), normalization (numeric data were normalized using step_normalize() to standardize the values), dummy encoding (categorical variables were encoded into dummy variables using step_dummy() to prepare them for model input), class imbalance handling (synthetic minority over-sampling technique {SMOTE} was applied using step_smote() to address the class imbalance in the outcome variable), zero variance removal (variables with zero variance were identified and removed using step_zv()), and correlation removal (correlated predictors were removed using step_corr() with a Spearman correlation threshold of 0.7 to reduce multicollinearity). The recipe was then applied to the training data using the prep() function, and the preprocessed data were extracted using the juice() function for model training.

Model Specification, Training, and Cross-Validation

Three machine learning models were employed to perform the classification task: Logistic Regression, Random Forest, and XGBoost. The Logistic Regression model was specified using the logistic_reg() function and fitted with a generalized linear model (GLM) engine in classification mode. The model was then incorporated into a workflow using the workflow() function; combined with the previously prepared recipe, the Random Forest model was specified using rand_forest() with the ranger engine. Feature importance was calculated using impurity-based measures. This model was similarly added to a workflow, which included the preprocessing recipe; the XGBoost model was specified using boost_tree() with the XGBoost engine in classification mode. Like the other models, the XGBoost model was incorporated into a workflow that included the same preprocessing steps. To evaluate the performance of the models and tune their hyperparameters, 10-fold cross-validation was performed on the training data. The vfold_cv() function was used to implement this, with stratification based on the outcome variable (case) to ensure that each fold was representative of the overall class distribution.

Model Fitting and Feature Importance Web Development

Each of the three models was fitted to the cross-validation folds using the fit_resamples() function. The models were evaluated based on multiple performance metrics, including accuracy, recall, precision, F1 score, kappa, area under the curve-receiver operating characteristics (AUC-ROC), sensitivity, and specificity. The predictions generated by each model were compared to the true labels using confusion matrices. ROC curves were also generated to visualize and assess the models' performance. After the models were evaluated, the performance metrics were collected using the collect_metrics() function. These metrics were then compared across all three models using visualizations such as bar plots and ROC curves. The model with the highest mean AUC-ROC and F1 score was selected as the best-performing model for this study.

For the Random Forest and XGBoost models, feature importance was assessed using impurity-based measures. The top features were visualized using the vip() package to provide insights into the most influential predictors in the models. Additionally, SHapley Additive exPlanations (SHAP) values were calculated using the shapper package. These values helped interpret the contributions of individual features to the model predictions and were visualized for individual cases to enhance the understanding of the models' decision-making processes.

Based on the best-performing model, a web-based interface was developed using RShiny(). This interface allows clinicians and researchers to input patient data and receive real-time predictions of ischemic stroke risk. The interface provides visualizations of feature importance and SHAP values, offering transparency into the model's decision-making process. The tool is designed to be user-friendly, enabling seamless integration into clinical workflows and facilitating personalized risk assessment for ACS patients.

## Results

Study population

The study collected data from EMRs of patients diagnosed with ACS at Dr. Sardjito Hospital between 2018 and 2022. A total of 12,358 visits corresponding to 4,966 unique ACS patients were identified. After excluding 117 patients who had a prior history of stroke or lacked follow-up data, the final study cohort consisted of 4,789 patients.

The demographic data and comorbidities were collected during the initial diagnosis of ACS. If a patient experienced more than one type of ACS within a year, the diagnosis with the highest severity was selected, based on the hierarchy of STEMI, NSTEMI, and unstable angina. Ischemic stroke occurrences were identified from follow-up visits within one year post-ACS, using relevant ICD-10 codes.

Characteristics of study subjects

Among the 4,789 ACS patients included in the study, 212 (4.4%) experienced ischemic stroke within one year. This incidence rate is consistent with findings from previous studies, such as the study by Ulvenstam et al. (2014), which reported a 4.1% stroke rate in ACS patients in Sweden over the first year post-diagnosis [12].

The baseline characteristics of the study population revealed several key insights related to gender, age, ACS type, and comorbidities (Table 1). The study population was predominantly men, comprising 77.9% of the total participants. However, there was no statistically significant difference in the risk of ischemic stroke between male and female patients (p = 0.472). Age was a significant factor, with patients aged 60 years or older showing a higher likelihood of experiencing ischemic stroke. Specifically, 66.5% of the stroke group were aged 60 or older, compared to 33.5% who were younger than 60, and this difference was statistically significant (p < 0.001).

**Table 1:** Comparison of Clinical Characteristics Between Ischemic Stroke and Non-ischemic Stroke Patients *P-value of less than 0.05, which means the result is statistically significant ACS, acute coronary syndrome; STEMI, ST-segment elevation myocardial infarction; NSTEMI, non-ST-segment elevation myocardial infarction; PCI, percutaneous coronary intervention; CABG, coronary artery bypass grafting

Characteristics	Ischemic Stroke (n = 212)	Non-ischemic Stroke (n = 4,577)	P-value
Gender, n (%)			
Female	42 (19.8)	1,014 (22.2)	0.472
Male	170 (80.2)	3,563 (77.8)	-
Age, n (%)			
≥60 years	141 (66.5)	2,316 (50.6)	<0.001*
<60 years	71 (33.5)	2,261 (49.4)	-
Type of ACS, n (%)			
STEMI	169 (79.7)	3,032 (66.2)	<0.001*
NSTEMI	36 (17.0)	916 (20.0)	-
Unstable angina	7 (3.3)	629 (13.7)	-
Atrial fibrillation, n (%)	43 (20.3)	411 (9.0)	<0.001*
Heart failure, n (%)	29 (13.7)	481 (10.5)	0.177
Hypertension, n (%)	124 (58.5)	2,173 (47.5)	0.002*
Dyslipidemia, n (%)	23 (10.8)	521 (11.4)	0.897
Diabetes mellitus, n (%)	93 (43.9)	1,429 (31.2)	<0.001*
Chronic kidney disease, n (%)	116 (54.7)	1,366 (29.8)	<0.001*
PCI, n (%)	118 (55.7)	2,132 (46.6)	0.012*
CABG, n (%)	0 (0.0)	7 (0.2)	1.000
Fibrinolysis, n (%)	8 (3.8)	156 (3.4)	0.926

The type of ACS also played a significant role in stroke risk. The majority of ischemic stroke cases occurred in patients with STEMI, accounting for 79.7% of the stroke group. This was followed by NSTEMI at 17.0% and unstable angina at 3.3%. The type of ACS was a statistically significant predictor of stroke, with a p-value of less than 0.001.

The presence of comorbidities was closely examined in relation to stroke risk. Atrial fibrillation was significantly more common in the stroke group, present in 20.3% of these patients, compared to 9.0% in the non-stroke group (p < 0.001). Heart failure was detected in 13.7% of stroke patients, slightly higher than the 10.5% observed in non-stroke patients, though this difference did not reach statistical significance (p = 0.177).

Hypertension was another critical factor, being more prevalent among stroke patients (58.5%) than those without stroke (47.5%), with a statistically significant difference (p = 0.002). Similarly, diabetes mellitus was more frequently observed in the stroke group, affecting 43.9% of these patients, compared to 31.2% of the non-stroke group (p < 0.001).

Chronic kidney disease was significantly more common in the stroke group, present in 54.7% of patients, compared to 29.8% in the non-stroke group (p < 0.001). Additionally, PCI was more frequently performed in stroke patients, with 55.7% undergoing the procedure, compared to 46.6% of non-stroke patients, and this difference was statistically significant (p = 0.012).

Machine learning model performance

**Figure 1:**
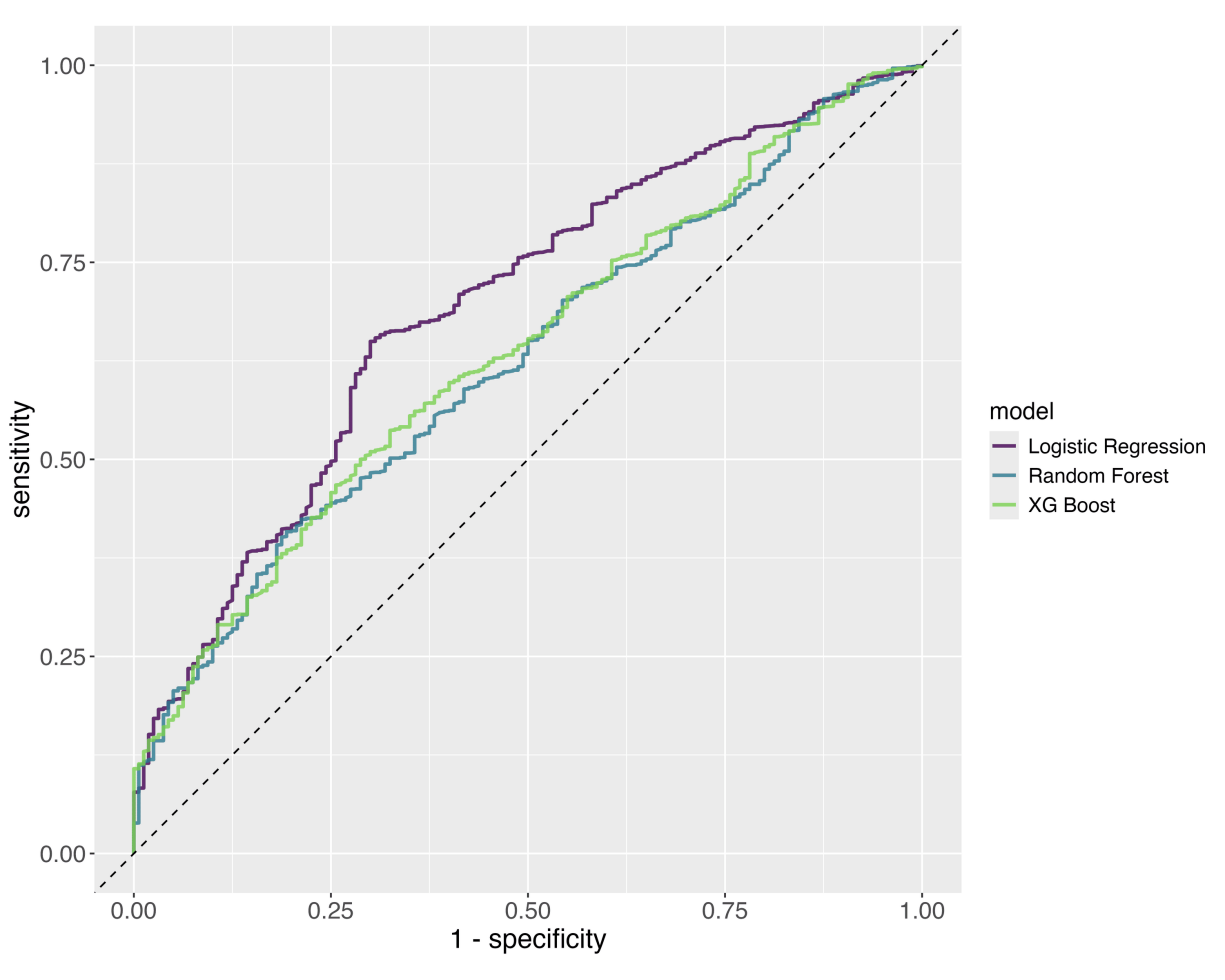
Receiver Operating Characteristic Curve of the Three Machine Learning Algorithms

This study employed three machine learning algorithms, Logistic Regression, Random Forest, and XGBoost, to predict ischemic stroke in patients with ACS. Each model was evaluated using key performance metrics, including precision, accuracy, sensitivity, specificity, and area under the curve-receiver operating characteristic (AUC-ROC) curve (Table 2 and Figure 1).

**Table 2:** Performance Metrics for Machine Learning Models

Machine Learning Model	Accuracy	Precision	Sensitivity	Specificity
Logistic Regression	0.70	0.95	0.65	0.70
Random Forest	0.90	0.94	0.94	0.12
XGBoost	0.90	0.94	0.95	0.12

Logistic Regression demonstrated a precision of 0.70, with an accuracy of 0.95. The model exhibited moderate sensitivity at 0.65 and a specificity of 0.70, with an AUC-ROC of 0.7. This indicates that while Logistic Regression was less sensitive in identifying true-positive cases, it maintained a balanced performance overall, effectively minimizing false positives. Random Forest achieved a higher precision of 0.90 and an accuracy of 0.94. The model's sensitivity was high at 0.94, but its specificity was notably low at 0.12. The AUC-ROC for Random Forest was 0.7, similar to Logistic Regression. The high sensitivity suggests that Random Forest was effective in identifying patients at risk for ischemic stroke, but the low specificity indicates a significant number of false positives, reducing the model's reliability. XGBoost performed similarly to Random Forest, with a precision of 0.90 and an accuracy of 0.94. The model's sensitivity was slightly higher at 0.95, but like Random Forest, it suffered from low specificity at 0.12. The AUC-ROC for XGBoost was also 0.7. This model, while highly sensitive, also produced a substantial number of false positives, leading to the potential overprediction of stroke risk.

Model comparison

The Random Forest and XGBoost models both demonstrated high sensitivity (0.94 and 0.95, respectively), indicating their strong ability to identify true-positive cases of ischemic stroke. However, this came at the cost of specificity, which was low for both models (0.12). The low specificity indicates that these models frequently misclassified non-stroke patients as being at risk, leading to a high number of false positives. This reduced the overall reliability of the predictions, making these models less ideal for clinical applications where false positives can lead to unnecessary interventions.

In contrast, the Logistic Regression model showed a more balanced performance, with moderate sensitivity (0.65) and higher specificity (0.70). This balance resulted in fewer false positives, making the predictions more reliable. Although the Logistic Regression model was less sensitive than the other two models, its higher specificity makes it a more suitable choice for scenarios where avoiding false positives is crucial. The AUC-ROC of 0.7 across all models indicates that while each model has its strengths, Logistic Regression offers the most balanced approach for predicting ischemic stroke in ACS patients, particularly in a clinical setting where both sensitivity and specificity are important.

The ROC curves shown in Figure 1 indicated that Logistic Regression provided the best overall performance, with a more balanced trade-off between sensitivity and specificity, making it the most suitable model for predicting ischemic stroke in this patient population.

Key predictors of ischemic stroke

**Figure 2:**
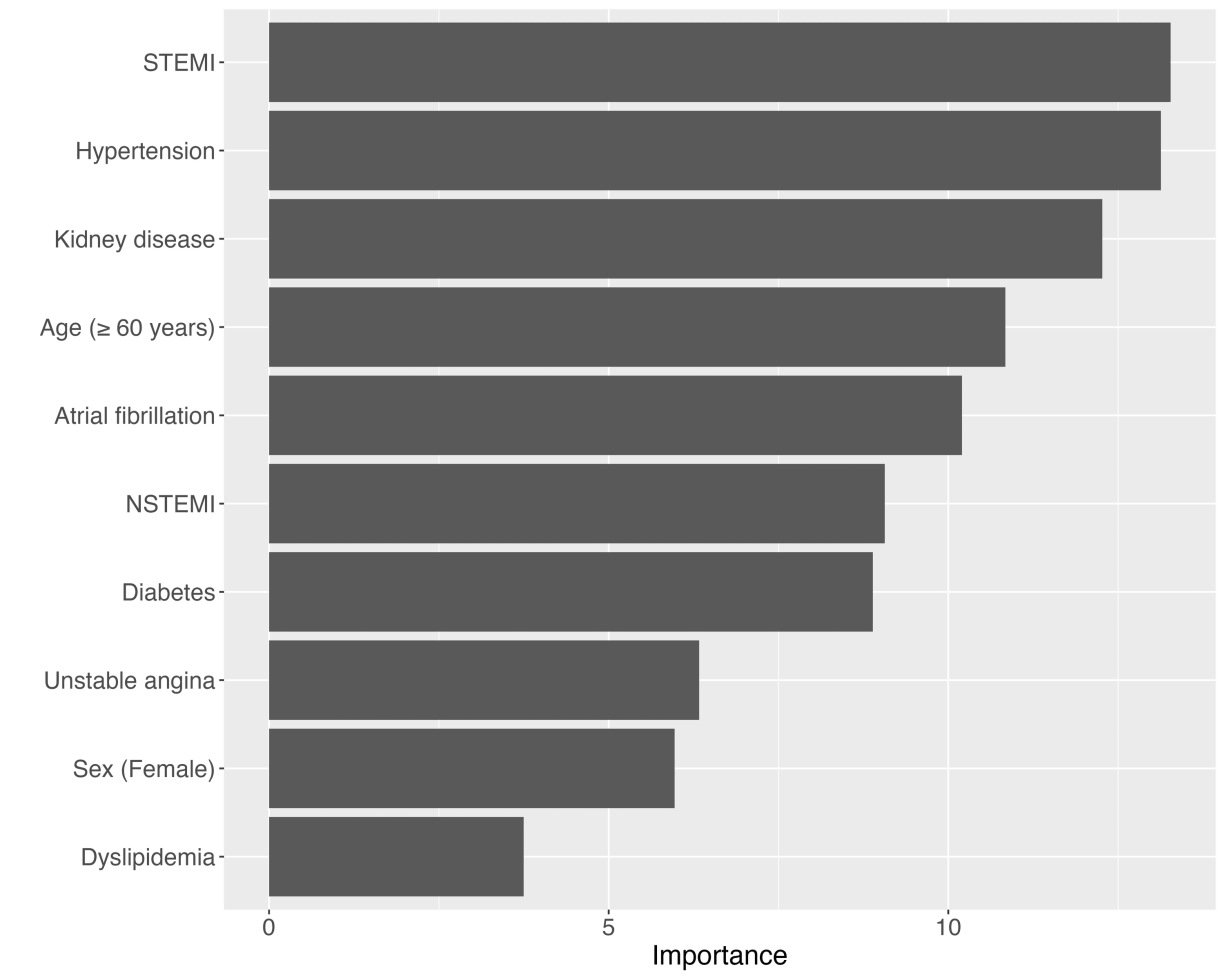
Top 10 Variables With the Highest Significance in Predicting Ischemic Stroke in Acute Coronary Syndrome Patients Based on the Logistic Regression Algorithm STEMI, ST-segment elevation myocardial infarction; NSTEMI, non-ST-segment elevation myocardial infarction

The Logistic Regression model used in this study highlighted 10 variables as the most significant predictors of ischemic stroke in patients with ACS. Among these, STEMI emerged as the most significant predictor, being strongly associated with an increased risk of ischemic stroke. This finding underscores the severity of STEMI, which is known to cause extensive myocardial damage, leading to a higher likelihood of thromboembolic events, including stroke. In this model, feature importance is quantified by the magnitude of each variable's coefficient, with larger absolute values indicating a stronger influence on predicting stroke risk (Figure 2).

Hypertension was another critical risk factor identified by the model, consistent with numerous studies that have linked elevated blood pressure to a heightened risk of stroke. Hypertension contributes to arterial damage, which not only increases the risk of myocardial infarction but also predisposes patients to cerebrovascular events.

Chronic kidney disease also stood out as a significant predictor of stroke, highlighting the crucial role that renal function plays in determining stroke risk following an ACS event. Impaired kidney function often leads to increased cardiovascular stress, systemic inflammation, and a pro-thrombotic state, all of which can elevate the risk of stroke.

Age was another important factor, with patients aged 60 years or older being at a significantly higher risk of ischemic stroke. This finding is consistent with the well-established understanding that age is a major risk factor for both cardiovascular and cerebrovascular events, making older patients particularly vulnerable.

Atrial fibrillation was identified as a major contributor to stroke risk in this population. This arrhythmia is well-known for its role in promoting the formation of blood clots, which can travel to the brain and cause a stroke, making it a critical factor in risk assessments.

NSTEMI was also associated with an increased risk of ischemic stroke, though to a lesser extent than STEMI. This highlights the need to consider different types of myocardial infarction when assessing stroke risk.

Diabetes mellitus further contributed to the risk of ischemic stroke in ACS patients. As a well-known risk factor for a range of cardiovascular complications, diabetes exacerbates the risk of both macrovascular and microvascular events, including stroke.

Female gender was another variable that emerged as a significant predictor of stroke risk. This finding aligns with some research suggesting that women may have a higher stroke risk following ACS, potentially due to differences in hormonal influences and cardiovascular physiology.

Finally, dyslipidemia was also identified as a significant predictor, contributing to the increased risk of stroke. The relationship between abnormal lipid levels and the development of atherosclerosis, which is a common underlying cause of ischemic strokes, reinforces the importance of managing dyslipidemia in ACS patients.

Taken together, these predictors provide a comprehensive understanding of the factors that contribute to ischemic stroke risk in ACS patients, emphasizing the need for careful risk stratification and targeted interventions in this population.

Development of a predictive model

**Figure 3:**
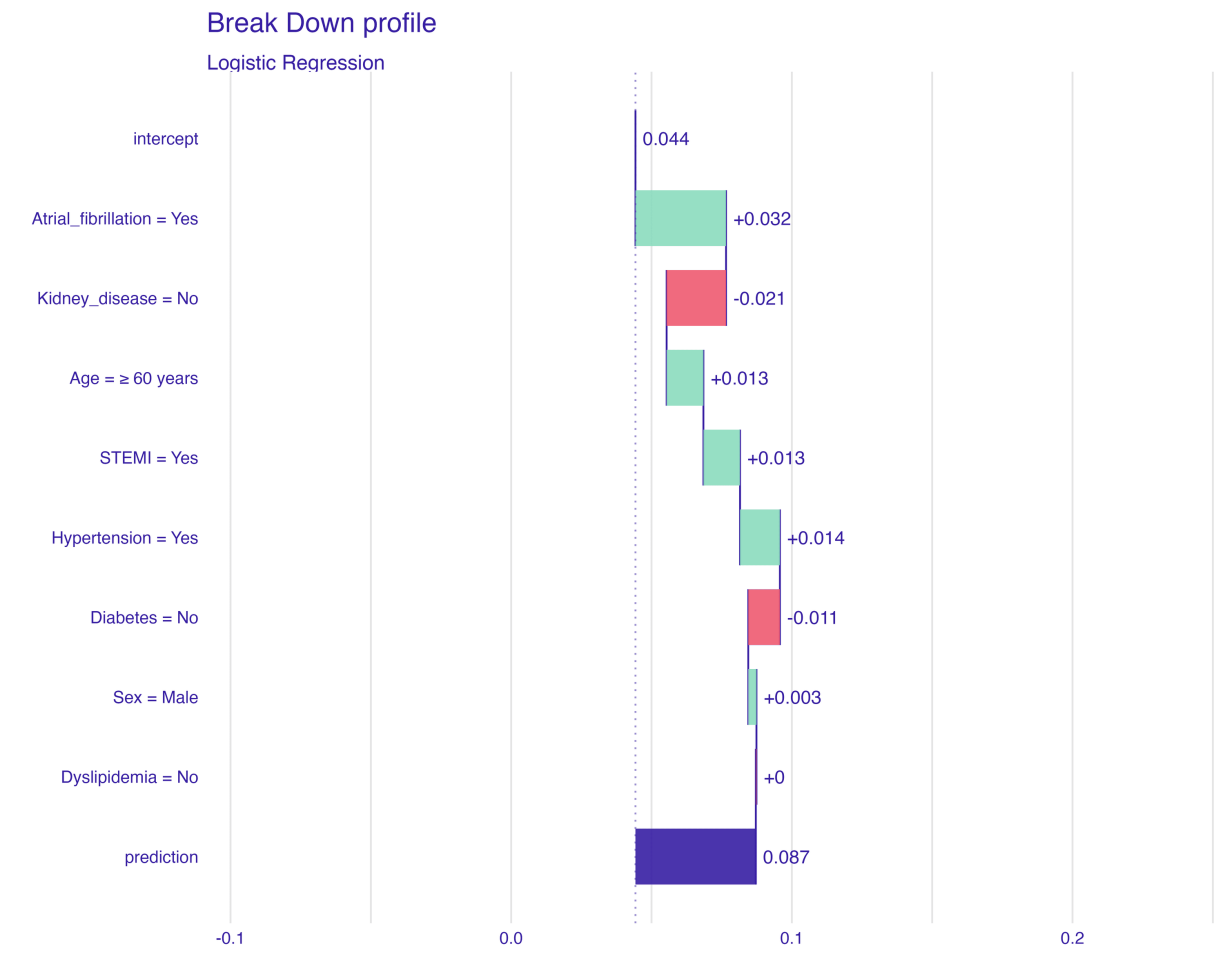
Simulation of Stroke Risk Prediction in Acute Coronary Syndrome Patients Using SHAP Plot SHAP, SHapley Additive exPlanations; STEMI, ST-segment elevation myocardial infarction

To aid clinical decision-making, the study proposes the development of a web-based tool using the Logistic Regression model (Figure 3). This tool will allow clinicians to input patient data and receive real-time predictions of stroke risk. The model's predictions will be visualized using SHAP plots to enhance interpretability, enabling better-informed therapeutic and preventive decisions in the management of ACS patients at risk of ischemic stroke.

For example, as illustrated in Figure 3, the breakdown plot shows how different features contribute to the final prediction for an individual patient with the following characteristics: atrial fibrillation, yes; kidney disease, no; age of ≥60 years; STEMI, yes; hypertension, yes; diabetes, no; sex, male; and dyslipidemia, no.

In this case, the model predicts a 0.087 probability, meaning there is an 8.7% chance that the patient will have an ischemic stroke. Positive contributors to the risk include factors such as atrial fibrillation (+0.032), STEMI (+0.013), and hypertension (+0.014), while other factors such as the absence of kidney disease (-0.021) and diabetes (-0.011) reduce the predicted risk. The intercept starts the baseline prediction at 0.044, and each factor adjusts the final prediction, resulting in the 8.7% risk. This detailed breakdown helps clinicians understand how specific patient characteristics influence the model's prediction, allowing for more personalized risk management.

## Discussion

The findings of this study underscore the significant role that ML models can play in predicting ischemic stroke in patients with acute coronary syndrome (ACS). Through the application of Logistic Regression, Random Forest, and XGBoost algorithms, the study identified key predictors of ischemic stroke, including older age, atrial fibrillation, STEMI, hypertension, chronic kidney disease, and diabetes mellitus. These findings are consistent with existing literature, which has established these factors as major contributors to stroke risk in post-ACS patients [5].

For instance, previous studies, such as those by Yaghi et al. (2016) [13] and Albaeni et al. (2020) [14], have highlighted the elevated stroke risk associated with conditions such as atrial fibrillation and STEMI. These studies corroborate our findings that patients with STEMI are at a higher risk of ischemic stroke compared to those with unstable angina [3,15]. Moreover, the study identifies hypertension and chronic kidney disease as significant predictors [16,17].

The use of machine learning in this study provided a more nuanced understanding of these relationships compared to traditional statistical methods. Logistic Regression, while offering a balanced trade-off between sensitivity and specificity, demonstrated the ability to predict stroke outcomes with a reasonable level of accuracy. On the other hand, Random Forest and XGBoost, despite their higher sensitivity, exhibited lower specificity, leading to a higher rate of false positives. This difference highlights the importance of model selection based on the specific clinical context and the desired balance between sensitivity and specificity [17,18].

In summary, the integration of ML techniques in predicting ischemic stroke risk among ACS patients not only reinforces established clinical knowledge but also enhances predictive accuracy, thereby potentially improving patient management strategies.

The results of this study have several important implications for clinical practice. The ability to accurately predict ischemic stroke in patients with ACS can significantly enhance risk stratification, allowing clinicians to identify high-risk individuals who may benefit from more aggressive monitoring and preventive strategies. For instance, patients identified as high risk by the machine learning models could be candidates for early anticoagulation therapy, closer surveillance, or more intensive management of comorbid conditions such as hypertension and atrial fibrillation [19,20].

The identification of key predictors, such as older age, atrial fibrillation, STEMI, hypertension, chronic kidney disease, and diabetes mellitus, aligns with existing literature that emphasizes the importance of these factors in stroke risk among post-ACS patients [3,21]. Studies have consistently highlighted the elevated stroke risk associated with conditions such as atrial fibrillation and STEMI, underscoring the necessity for targeted interventions in these vulnerable populations [13,14] and reinforcing the need for targeted interventions in these populations [22,23]. The study's findings suggest that clinicians should prioritize monitoring and management strategies for patients exhibiting these risk factors, potentially improving patient outcomes through timely interventions.

Furthermore, the development of a web-based tool based on the Logistic Regression model, as proposed in this study, could provide a practical and accessible resource for clinicians. Such a tool could facilitate real-time risk assessment in clinical settings, enabling personalized treatment plans that are tailored to the specific risk profile of each patient [24]. The incorporation of SHapley Additive exPlanations (SHAP) plots within this tool offers transparency in decision-making, allowing clinicians to understand the contribution of individual risk factors to the overall stroke risk [25]. This transparency is crucial for fostering clinician trust in the model's predictions and for enhancing shared decision-making with patients.

This study is not without its limitations. First, the use of data from a single center (Dr. Sardjito Hospital) may limit the generalizability of the findings to other populations and settings. The patient population at this center may have specific characteristics that differ from those in other regions or healthcare systems, which could influence the model's performance when applied elsewhere. Second, the potential biases inherent in EMR data, such as inaccuracies in coding or missing data, could have impacted the study's results. The reliance on ICD-10 codes for identifying comorbidities and outcomes, without an external validation of these codes, introduces a risk of misclassification. Additionally, the imbalance in the number of patients with and without ischemic stroke may have affected the performance of the machine learning models, particularly in terms of specificity. While techniques such as SMOTE were employed to address this imbalance, future studies with more balanced datasets could provide further insights.

Finally, the study did not consider certain clinical variables, such as laboratory results or the use of specific pharmacological interventions, which could potentially influence stroke risk. The inclusion of these variables in future analyses could enhance the predictive power of the models.

## Conclusions

This study illustrates the effective development and validation of a predictive model for ischemic stroke in patients with ACS utilizing machine learning methodologies, particularly Logistic Regression. The model utilizes an extensive dataset from electronic medical records to identify significant clinical characteristics that influence stroke risk, such as atrial fibrillation, STEMI, age, hypertension, and kidney disease. These results correspond with established clinical knowledge and offer a quantitative instrument for real-time risk evaluation.

This study establishes a robust basis for employing predictive analytics in the prevention and management of ischemic stroke in ACS patients, potentially enhancing patient outcomes through timely and informed interventions.
